# An experience sampling study on the links between daily teacher self-efficacy, need-supportive teaching and student intrinsic motivation

**DOI:** 10.3389/fpsyg.2023.1159108

**Published:** 2023-07-20

**Authors:** Elisa Kupers, Judith Loopers, Casper Albers, Alianne Bakker, Alexander Minnaert

**Affiliations:** ^1^Department of Inclusive and Special Needs Education, Faculty of Behavioral and Social Sciences, University of Groningen, Groningen, Netherlands; ^2^Department of Psychometrics and Statistics, Faculty of Behavioral and Social Sciences, University of Groningen, Groningen, Netherlands

**Keywords:** teacher self-efficacy, motivation, need-supportive teaching, diary study, experience sampling, teacher-student interaction

## Abstract

**Introduction:**

Why are some teachers more successful at motivating students than others? We know from previous literature that teachers’ self-efficacy relates to the extent in which they engage in need-supportive teaching in the classroom, which in turn relates to student intrinsic motivation. However, teachers’ self-efficacy is hypothesized to be dependent on their previous mastery experiences, e.g., of engaging students in the classroom. This “feedback loop” where the teacher not only influences the student but also the other way around, in a process unfolding over time, can only be investigated empirically with an intensive longitudinal design. This is precisely what we did in the current study.

**Methods:**

Secondary school teachers (*n* = 4) and students (*n* = 90) participated in an experience sampling study throughout one school year, resulting in a unique dataset with 48–59 repeated measurement points per class.

**Results:**

Visual exploration of the time series revealed that teacher self-efficacy can vary substantially from lesson to lesson, with characteristic patterns of stabilization and de-stabilization. We conducted Vector Autoregressive Analysis (VAR) for each of the four cases to test whether, and how, the variables relate to each other over time. We found an “overspill effect” for student motivation, meaning that students’ motivation in today’s lesson predicts their motivation in tomorrow’s lesson. Furthermore, in two cases we found that today’s student motivation predicts tomorrow’s teacher self-efficacy, but not the other way around.

## 1. Introduction

Why are some teachers more successful at motivating students than others? From the literature on student motivation, we know about the specific teacher strategies and behaviors that can contribute to students’ motivation and engagement in the classroom (Stroet et al., 2013; Olivier et al., 2021). However, this does not explain why some teachers manage to implement these strategies consistently while others struggle to engage students in their lessons. What is more, the extent to which teachers manage to motivate students might even vary from lesson to lesson. Just like students, teachers are agentic human beings and their actions, emotions and motivations emerge in the day-to-day practice of teaching and learning (Steenbeek and van Geert, 2013). Teaching and learning of both students and teachers are deeply interrelated: the teacher does not only influence the student, but also the other way around. Although recognized theoretically (e.g., Zee and Koomen, 2016; Woolfolk Hoy, 2021), this bi-directional influence is hardly ever the topic of empirical research. The aim of this study therefore is to investigate how day-to-day lesson experiences of teachers and students relate to each other over time.

An important element of teachers’ motivation for teaching is self-efficacy (Woolfolk Hoy, 2021). Self-efficacy is the extent to which a person expects to be successful in implementing behaviors that lead to certain (desirable) outcomes (Bandura, 1977). For teachers this refers to their personal convictions that they have control over the learning and behavioral outcomes of their students through their own teaching practices (Tschannen-Moran et al., 1998). Although mostly researched as a general, stable trait, more and more studies reveal that teacher self-efficacy is more specific and more variable than previously thought. First, self-efficacy turns out to be specific to certain domains: a teacher might feel well able to control students’ learning outcomes, but might feel less secure about their impact on students’ well-being or motivation (Skaalvik and Skaalvik, 2007; Zee et al., 2016). Second, self-efficacy is also student-specific: teachers in particular experience less self-efficacy in relation to students with externalizing behavior, compared to more typically developing students (Zee et al., 2017). Third, although teacher self-efficacy is usually measured cross-sectionally at only one or two points in time, we know from studies on self-efficacy in other populations that self-efficacy can vary substantially from day to day or even from moment to moment (Bouchard et al., 2007; Weikel et al., 2017). For instance, in an intensive longitudinal study on self-esteem (a construct related to self-efficacy) revealed not only that self-esteem varies across multiple timepoints a day, but also that the longitudinal pattern of self-esteem shows a particular fractal pattern known as “pink noise”, a specific type of patterned fluctuations (contrary to white noise – a more random pattern of fluctuations) which is a characteristic in all kinds of complex dynamical systems existing in nature (for instance in sounds and heart rate) with a feedback loop (Delignières et al., 2004). Therefore, we expect teacher self-efficacy to depend not only on factors relating to each individual lesson, but also to be dependent on its own history.

Self-efficacy is a central construct in relating both to outcomes on the teacher, as well as on the student level. For teachers, this link is relatively straightforward. Self-efficacy is considered an important condition for experiencing motivation, and in turn, experiencing motivation for one’s work is an important factor in well-being. Previous studies indeed show that self-efficacy relates positively to various indicators of teacher well-being (including job satisfaction, engagement (Wang, 2021) and the absence of stress and burnout) [see Zee and Koomen (2016) for a systematic overview].

The relation between teacher-self-efficacy and student motivation is more complex and indirect (Woolfolk Hoy, 2021). Although it is assumed that aspects of teacher motivation matter for student motivation, this assumption remains largely uninvestigated (Daumiller et al., 2021). In the literature on teacher self-efficacy, “only about 3% of the available studies on teacher self-efficacy has focused on the link with student outcomes” (Lauermann and Berger, 2021). The assumed path from teacher self-efficacy to student motivation moves via teacher behaviors and instructional strategies. When one experiences high self-efficacy, one is more inclined to use effective coping behavior in a situation, and will also be able to sustain this behavior in the face of challenges (Bandura, 1977). There is some (mainly cross-sectional) evidence that teachers with high self-efficacy do things differently in class (Zee and Koomen, 2016): they are more likely to support their students’ learning by offering structure to the learning material and the learning process. At the same time, they also show more affective involvement. Not coincidentally, these behaviors are at the core of what literature describes as “need-supportive teaching”. Need supportive teaching consists of practices by which teachers foster students’ sense of autonomy, competence and relatedness (Stroet et al., 2013): three basic psychological needs according to the Self-Determination Theory (Deci and Ryan, 1985; Deci et al., 1991). Self-Determination Theory further distinguishes between different types of motivation, which differ in the extent to which they are controlled by external influences (such as rewards and punishments, but also the expectations of others). Autonomous types of motivation are characterized by minimal to no external control. Autonomously motivated students learn out of either an agentic feeling of purpose and meaningfulness (identified motivation) or out of sheer interest and fascinitation with the learning content (intrinsic motivation). The fulfillment of students’ basic psychological needs is considered a prerequisite for experiencing more autonomous types of motivation, especially intrinsic motivation (Deci et al., 1991). A sense of autonomy is fostered by autonomy-supportive teaching practices, which include letting students make meaningful choices and responding to student initiatives and questions. The need for competence is met if the teacher provides structure through clear learning goals, informative feedback and solid classroom organization. Considering the need for relatedness, both relatedness between teacher and students is important, as well as relatedness amongst the students. The teacher can support the first kind of relatedness by showing involvement through being genuinely interested in their students and getting to know them well (Stroet et al., 2013).

If teacher self-efficacy has such important consequences, one might ask where it stems from. In self-efficacy theory it is explained how self-efficacy emerges from previous experiences of having control over one’s environment: the experience of mastery (Bandura, 1977). One can easily imagine how higher or lower levels of self-efficacy emerge in a positive feedback loop or a negative “vicious cycle”. A teacher with a high level of self-efficacy feels confident enough to implement structure in her lesson and shows affective involvement to the students. This leads to positively engaged students, and in turn to desired outcomes: a positive class climate and intrinsically motivated students who meet their learning goals. This successful experience leads in turn to the teacher experiencing that her actions have the desired consequence; a high level of self-efficacy which in the next lesson leads to a higher chance of more mastery experiences due to effective teaching practices, and so on. A teacher who is confronted with unmotivated students and a chaotic course of the lesson might conclude consciously or subconsciously that she has limited control over the students’ motivation, resorting to more controlling and less need-supportive teaching, which aggravates the problems with students’ behavior, more feelings of helplessness on the side of the teacher, and so on.

Intuitively comprehensible and theoretically founded as these developmental trajectories might be, these mechanisms have never been fully tested empirically. By far most empirical research on teacher self-efficacy relating to student outcomes has been conducted cross-sectionally with one or at most a handful of measurement points. One of the reasons why this is problematic is because of what Molenaar (2004) calls the “ergodicity assumption”. This is the assumption that relations between variables on a group level (where teacher self-efficacy can be said to predict student motivation, meaning teachers with a high sense of self-efficacy on average have students with higher levels of (intrinsic) motivation) can be generalized to the individual level (when a teacher feels self-efficacious in a certain lesson, this will impact his teaching and consequently his students’ intrinsic motivation in the next lesson). Empirical research in educational psychology has shown that the ergodicity assumption not always holds for mechanisms pertaining to learning and motivation (Murayama et al., 2017). This means that research with dense intra-individual data is needed, capturing teacher self-efficacy, need-supportive teaching and student intrinsic motivation from lesson to lesson over longer periods of time. Mobile technology, where teachers and students fill out short questionnaires on their phone after each lesson, hold tremendous potential for gathering such data (Murayama et al., 2017). This is also known as experience sampling (ESM) or ecological momentary assessment (EMA) in the literature. ESM research has a long tradition in (mostly clinical) psychology, but the application to educational psychology is a fairly new, yet growing, area of interest.

The aim of this multiple case study is to zoom in on lesson-to-lesson development of teacher self-efficacy, to first find out to what extent self-efficacy is indeed variable on a day-to-day level. Consequently, we aim to investigate how these lesson-to-lesson changes in teacher self-efficacy relate to need-supportive teaching and students’ intrinsic motivation. To the best of our knowledge, this is the first study with an intensive longitudinal design (intensive meaning a design that allows for time series analysis) that links teacher and student experiences. This study thereby provides a unique, more detailed look at the dynamics of self-efficacy within teachers and in interactions with their students, thereby increasing our fundamental knowledge on how self-efficacy emerges as a socially situated construct over time. We aim to answer the following research questions:

1. To what extent does teacher self-efficacy vary from lesson to lesson and over the course of one school year?
2. How do teacher self-efficacy, need-supportive teaching, and student intrinsic motivation relate to each other over time?

## 2. Methods

### 2.1. Design

In order to capture teacher self-efficacy as a momentary state which emerges out of fleeting, daily experiences of teachers and students in the classroom, we used an intensive longitudinal design. The design was longitudinal in the sense that it spanned the large part of one school year (20 weeks) and intensive in the sense that we aimed to gather experiences of teachers and one of their classes after every lesson they had together in these 20 weeks. Depending on the exact course schedule, this resulted in 40–60 repeated measurements per teacher/ class.

### 2.2. Participants

The participants in the current multiple case study were four secondary school teachers teachers (two mathematics and two native language teachers) and 90 of their students (49% males). Table 1 lists the background characteristics of the four participating teachers.

**Table 1:** Overview background characteristics of the four cases (teachers).

Teacher	Age	Gender	Subject	Years of experience
A	41–50	Female	Native language	20 + years
B	21–30	Male	Math	0–5 years
C	31–40	Female	Native language	6–10 years
D	31–40	Female	Math	11–15 years

These participants were drawn from a larger sample of 13 teachers and 255 students. The four cases were selected from the larger sample on the basis of the number of measurements they completed. The teachers (and their students) with the most complete dataset were selected. This was done to allow an in-depth analysis of the intensive longitudinal data with minimal bias due to missing data. The teachers included in the larger study were recruited via the professional network of the researchers, earlier research collaborations and via social media. We specifically recruited teachers that taught English, Dutch (native language) or mathematics to second year students in secondary vocational education. These inclusion criteria were set because language and math are core subjects that usually have a similar setup of the lesson (a combination of teacher explicit instruction and independent seat work). We included only teachers with second year classes to make sure that classes were comparable and differences in student age and achievement level played a minimal role. The participating teachers then asked their second-year students to participate. Teachers participated in principle with one of their classes; one teacher participated with two classes and two classes each participated with two teachers. Prior to the start of the study, teachers, students, and the students’ parents signed an informed consent form. The study procedure was approved by the Ethical Committee of the Department of Pedagogical and Educational Sciences, University of Groningen (d.d. October 8, 2018).

### 2.3. Procedure

Teachers and students filled out an experience sampling questionnaire via the online platform “u-can-act” (Blaauw et al., 2019) in an intensive longitudinal design of 20 weeks within one school year. After acquiring their schedule, the teachers and student received a text message on their phone straight after each of their lessons together (depending on the schedule, 2–3 times a week, resulting in 40–60 repeated measures per participant) with a link to their questionnaire. The questionnaire was designed so that it could be completed in less than 2 minutes to limit the workload of participants. To eliminate missing data as much as possible, teachers and students received points for each completed questionnaire (and additional points if they filled out at least three questionnaires in a row). At the end of the study, the points were translated into credit on a gift card. For some students, this could have been an incentive to submit a blank questionnaire, just for creadit on the gift card. To eliminate bias in our results, we considered questionnaires that were submitted within seconds and with all sliders left at the default value, as missing (as it takes 2 minutes to genuinely answer the questions).

### 2.4. Measurements

**Teacher self-efficacy** was measured in the teacher questionnaire with four items on a 6-point likert scale. The items were based on two scales of the TEIP (Teacher Efficacy for Inclusive Practice; Sharma et al., 2012): efficacy in managing behavior and efficacy in (inclusive) instructions. Examples of items were *“During the past lesson, I was able to give an alternative explanation when students did not understand something”* and *“During the past lesson, I was able to control disturbing behavior in the classroom”*. Cronbach’s alpha for this scale was *α* = 0.78.

**Teacher-reported (TR) need-supportive teaching** was measured in the teacher questionnaire with 8 items on a 6-point Likert scale pertaining to the teacher’s perceived ability to provide autonomy support, structure, involvement and support for relatedness between students (*α* = 0.82 for the total scale). The items were based on the Basic Psychological Need Satisfaction and Frustration scale (Chen et al., 2015). Examples of items are *“During the past lesson, I encouraged my students to ask questions.”* and *“During the past lesson, I showed interest in my students”*.

**Student intrinsic motivation** was measured in the student questionnaire with a scale of 7 items (*α* = 0.83). based on the interest/ enjoyment scale of the Intrinsic Motivation Inventory (Deci et al., 1994). Examples of items are *“I found the past lesson to be interesting/ boring”*. These items were measured on a 100-point scale with a slider. The student intrinsic motivation for each timepoint (lesson) was calculated as the average of the levels of intrinsic motivation of all students who had filled out the questionnaire in that particular lesson.

### 2.5. Analysis

In order to closely examine the development of teacher self-efficacy and TR need-supportive teaching over the school year, we first performed descriptive analysis (visual inspection of the time series and correlations between variables on the same measurement point). We defined missing data as points in the time series where a lesson had actually taken place, but either the student or the teacher questionnaires (or both) were missing. This means that school vacations, canceled lessons because of field trips or illness of the teacher etc. were not considered missing data. Missing data points in the time series were imputed five times via multiple imputation with the *mice* package (version 3.6.1) in R (Van Buuren and Groothuis-Oudshoorn, 2011. In order to test the links between teacher self-efficacy, TR need-supportive teaching and student intrinsic motivation over time, we then performed vector autoregression analyses (one regression model for each of the four cases) with the *vars* package (version 1.5–3) in R (Pfaff, 2008). Vector autoregression analysis is a particular form of regression analysis suited especially for time series and tests the relationship between variables over time (Zivot and Wang, 2006). In VAR models, contrary to regular regression analysis, there is no distinction between dependent and independent variables. The collection (vector) of variables at t_−1_ are used to predict the values of those variables at t. This makes the analysis technique suited for testing conceptual models where constructs (in this case, teacher self-efficacy, need-supportive teaching and student intrinsic motivation) potentially influence *each other over time*.

## 3. Results

### 3.1. Changes in teacher self-efficacy over time

**Figure 1:**
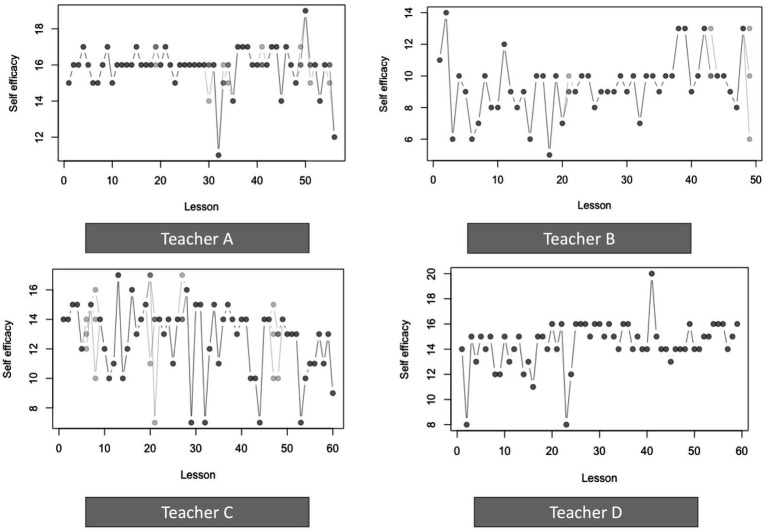
Time series of self-efficacy for teachers A, B, C, and D. Imputed values shown in lighter shades.

Figure 1 shows the values of each of the four teachers’ values of self-efficacy over time. As is clear from the figures, self-efficacy varies from lesson to lesson, though the lesson-to-lesson variability is more pronounced in some teachers than in others, and that patterns of variability and stability differ between teachers. Teacher A shows a pattern with relative stability (limited variability) at first and a sudden increase in variability after the 30th lesson. Teacher D shows a similar pattern in reverse: high variability in the first half of the time series and more stability after the 30th lesson. Teacher B and C (both teachers with <10 years of experience) show more variability in self-efficacy from lesson to lesson.

### 3.2. Correlations between self-efficacy, need-supportive teaching and student intrinsic motivation within lessons

Several things stand out in Table 2. First, when we look at correlations between variables at the same time point for each of the teachers, we see that the correlations between teacher self-efficacy and the different dimensions of TR need-supportive teaching are in general substantial, but also differ for each of the four teachers. High self-efficacy corresponds with high TR levels of need-supportive teaching, but the structure of these correlations is different for the different teachers. For teacher A, self-efficacy relates especially to self-reported provision of structure and involvement, and to a lesser but still substantial extent to the other dimensions of need-supportive teaching. With teacher B, the correlations between self-efficacy and TR need-supportive are generally lower. For teachers C and D, there is a strong relation between self-efficacy on the one hand and autonomy support, structure and involvement on the other hand, and to a lesser extent to the support of peer involvement. The different dimensions of TR need-supportive teaching also correlate with each other.

**Table 2:** Correlations between variables within the same lesson, for the four cases.

		Self-efficacy	Structure	Autonomy support	Involvement	Involvement support (peers)	Student intrinsic motivation
Teacher A	Self-efficacy	1					
	Structure	0.78**	1				
	Aut. Supp.	0.39**	0.43**	1			
	Involvement	0.70**	0.50**	0.41**	1		
	Inv. Supp. P.	0.56**	0.43**	0.40**	0.55**	1	
	Intr. Motivation	0.08	−0.13	0.37*	0.17	0.15	1
Teacher B	Self-efficacy	1					
	Structure	−0.02	1				
	Aut. supp	0.33*	0.08	1			
	Involvement	0.28	0.25	0.26	1		
	Inv. Supp. P.	−0.28	0.24	0.13	0.14	1	
	Intr. Motivation	0.36*	−0.05	0.10	0.23	0.25	1
Teacher C	Self-efficacy	1					
	Structure	0.74**	1				
	Aut. Supp.	0.69**	0.52**	1			
	Involvement	0.80**	0.61**	0.71**	1		
	Inv. Supp. P.	0.51**	0.30*	0.30*	0.43**	1	
	Intr. Motivation	−0.01	−0.12	−0.02	−0.02	0.02	1
Teacher D	Self-efficacy	1					
	Structure	0.60**	1				
	Aut. supp	0.69**	0.60**	1			
	Involvement	0.73**	0.69**	0.69**	1		
	Inv. Supp. P.	0.45**	0.12	0.33*	0.37**	1	
	Intr. Motivation	0.02	−0.04	−0.02	0.06	−0.03	1

* Correlation is significant at *p* = 0.05 (2-tailed). ** Correlation is significant at *p* < 0.01 (2-tailed).

Concerning the relationship of the teacher variables with student intrinsic motivation within the same lesson, for teacher C and D the correlations are close to zero. For teacher A, there is a moderate correlation between teacher-reported autonomy support and student intrinsic motivation, and for teacher B a moderate correlation between teacher self-efficacy and student intrinsic motivation.

### 3.3. Relations between variables over lessons

In order to test the relations between variables over time, we estimated four VAR models, one model for each of the four classes. Included variables were teacher self-efficacy, TR need-supportive teaching (the sum of autonomy support, structure, and the two involvement variables), and student-reported intrinsic motivation. We chose to use the sum score of TR need-supportive teaching because the length of the time series (48–56 in the four cases) limits the number of variables one can include. For all four models, the optimal lag was 1 (using the BIC criterion). Table 3 shows the results for the check of the stationarity assumption of the time series, tested with an Augmented Dicky Fuller test.

**Table 3:** Frequency of significant results of the augmented Dicky Fuller test for stationarity.

	Self-efficacy	TR Need-supportive teaching	Student intrinsic motivation
Teacher A	0/5*	1/5*	5/5
Teacher B	5/5	5/5	0/5*
Teacher C	5/5	5/5	0/5*
Teacher D	5/5	5/5	3/5

Each test is run 5 times, one time for each imputation. ^*^variables where the stationarity-assumption is most likely violated.

**Figure 2:**
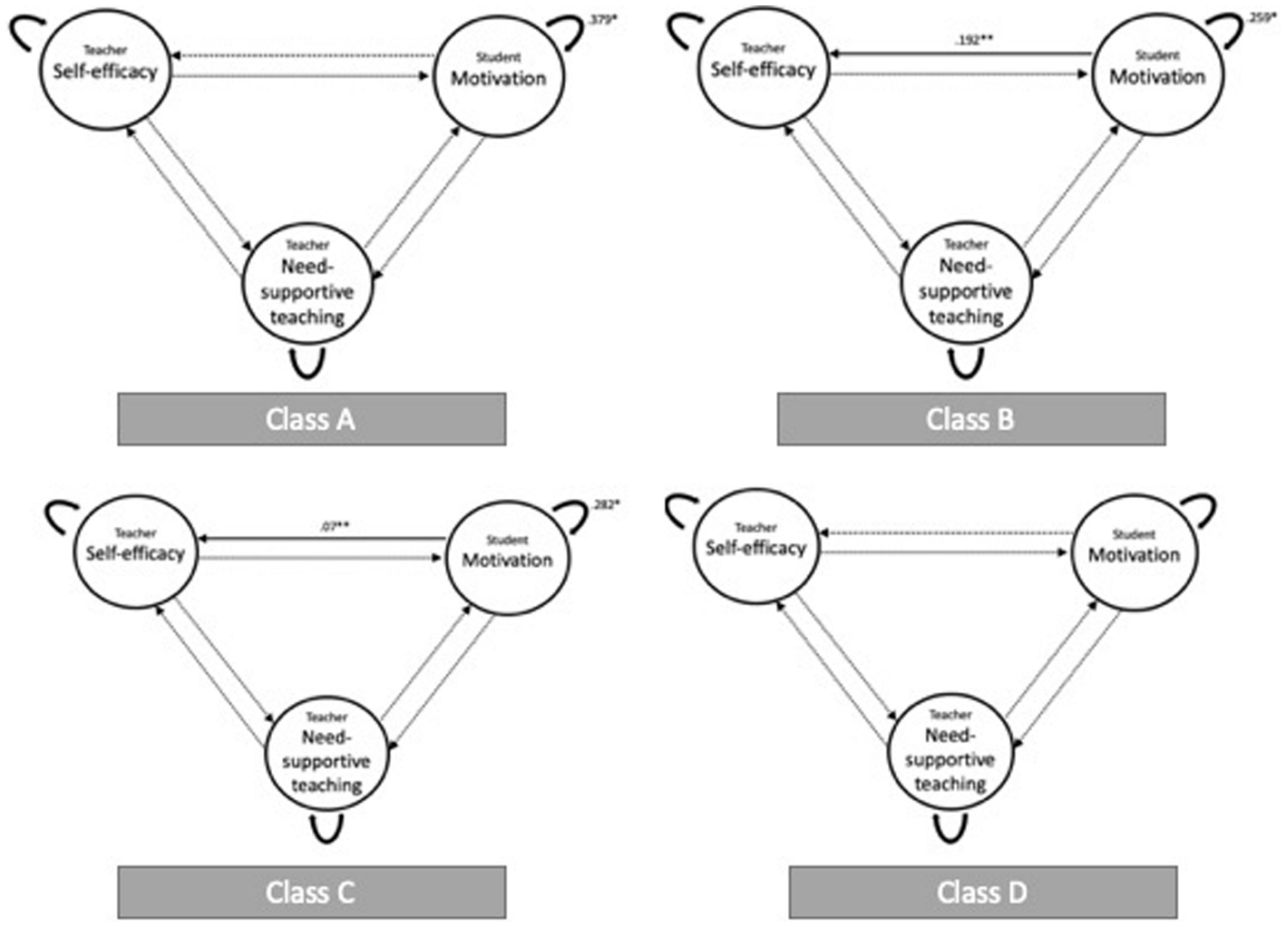
Summary of VAR results for the four classes. Dotted lines represent non-significant relations.

Figure 2 summarizes the results of the VAR models for each of the four classes visually. Each of the models’ coefficients (mean values over the multiple imputations were calculated) are given in Table 4. In three out of four classes, there is a significant relation between student intrinsic motivation at y*_t_* and y*_t + 1_*. In two out of the four classes, the relation between student intrinsic motivation at y*_t_* and teacher self-efficacy at y*_t + 1_* is also significant. Class D is the only case where none of the relations between variables were significant.

**Table 4:** Coefficients (mean values over all imputations) of the four VAR models.

	Self-efficacy*_t_*	TR Need Supp. Teaching*_t_*	Intr. Motivation*_t_*	Self-efficacy*_t_*	TR Need Supp. Teaching*_t_*	Intr. Motivation*_t_*
	Teacher A			Teacher B		
Self-efficacy*_t-1_*	−0.099	−0.349	−0.522	−0.107	−0.156	0.547
TR Need Supp. Teaching*_t-1_*	0.086	0,125	0.464	−0.007	0.139	−0.062
Intr. Motivation*_t-1_*	−0.015	0.011	0.379*	0.192*	0.142	0.259**
	Teacher C			Teacher D		
Self-efficacy*_t-1_*	0.253	0.59	0.743	0.077	0.089	−0.270
TR Need Supp. Teaching*_t-1_*	−0.157	−0.326	−0.156	0.032	0.110	0.274
Intr. Motivation*_t-1_*	0.070**	0.112	0.282*	−0.051	−0.149	0.047

*p < 0.05 in 5/5 imputations; **p < 0.05 in 3/5 imputations.

## 4. Discussion

The causes and consequences of teacher self-efficacy, and the possible bidirectional relations between daily teacher and student experiences can be substantiated theoretically but are rarely studied empirically. Therefore, the aim of this study was to investigate the interplay between teacher self-efficacy, need-supportive teaching, and student intrinsic motivation over time. We used an intensive longitudinal multiple case study design in which we could link the lesson-to-lesson experiences of teachers and students.

We found that teacher self-efficacy is not a stable trait, but instead varies from lesson to lesson, even when teaching the same class. Although longitudinal changes in teacher self-efficacy have been reported before (i.e., von Suchodoletz et al., 2018; Dursun, 2019), the unique intensive longitudinal design of this study allowed us to get a much more fine-grained picture of day to day variability in teacher self-efficacy. This observed variability is more pronounced in some teachers than in others. The four cases present three types of variability in self-efficacy: one pattern of stabilization, one pattern of de-stabilization and one pattern of overall lesson-to-lesson variability in self-efficacy. We did not see clear increases or decreases in self-efficacy over the course of the school year for any of the four teachers.

What is the meaning of these patterns in variability of teachers’ self-efficacy? Traditionally, variation around a mean is interpreted at least partially as measurement error around the “true mean” of any latent psychological variable. From the perspective of complexity science, however, which is also gaining terrain in educational psychology during the past decades (Koopmans and Stamovlasis, 2016; Larsen-Freeman, 2016), intra-individual variability is an important source of information. Basic psychological properties such as experienced emotions, but also higher order constructs such as attitudes and self-esteem vary on a day-to-day or even moment-to-moment basis. Like other natural systems, the pattern and degree of variability potentially hold information that aggregated scores of “general” self-efficacy do not. For instance, high levels of fluctuations in experienced emotions can be linked to an increased risk for developing psychological problems (Houben and Kuppens, 2020). In line with our findings, changes in complex dynamical systems are often non-linear, and destabilization of patterns (characterized by a sudden increase in variability, similar to what we found in one of the four cases) is often associated with qualitative change or the forming of new attractor states (see Evans and Larsen-Freeman, 2020 for an empirical example in the domain of L2 learning). Studies focusing explicitly on intra-indiviudal variability in education are scarce to date. In explorative studies, Mainhard et al. (2012) found higher levels of variability in teacher-student interactions of negative quality. More research is needed, especially research with intensive longitudinal designs, to clarify what the role of variability in teaching and learning entails.

We found that within lessons, there were relationships between especially the teacher-reported variables (self-efficacy and need-supportive teaching), but that the strength and pattern of these relationships differed for each of the four teachers. This latter finding implies that indeed, as Molenaar (2004) suggested, relationships between variables found in cross-sectional research at the group level cannot be fully generalized to the individuals in that group. One might also wonder about the conceptual overlap between the variables. For self-efficacy, teachers reported whether they felt they were able to meet certain needs of students or felt able to achieve certain outcomes with their students during the past lesson. For need-supportive teaching, teachers reported on what they did in terms of need-supportive teaching during that lesson. Because self-efficacy is reported retrospective (based on the lesson they just gave), there might be some conceptual overlap between the measured variables (teachers might think: apparently I was able to do this, *because I just did it*).

The relations between the variables over time were partly in line with our expectations. First, student intrinsic motivation is significantly autocorrelated in all four classes, which means that the intrinsic motivation of students tomorrow can be predicted based on their motivation today. In the psychological literature with intensive longitudinal data, this phenomenon is known as inertia: the tendency of people’s experienced emotions to spill over from one moment to the next (Koval et al., 2016). Based on our findings, this seems to be true for intrinsic motivation as well: students walk into the class of a particular teacher teaching a particular subject with expectations (positive or negative) and experiences of the previous lesson(s). That also means that intrinsic motivation could be resistant to change. The fact that we found this for the mean student intrinsic motivation in each lesson is remarkable since this was based on the mean of all students who filled out the questionnaire for that particular lesson only, which means that the mean intrinsic motivation can be based on the experienced intrinsic motivation of (partly) different students. When the intrinsic motivation would have been based on the reports of the same students, the relationship could probably have been even stronger.

Contrary to our expectations, we did not find significant autocorrelations for neither teacher self-efficacy nor for teacher-reported need-supportive teaching. Based on the literature we would have expected an effect especially for self-efficacy, considering the theoretical foundation of the “feedback loop” where positive or negative self-efficacy has a tendency to reinforce itself. This has been found empirically in previous studies for self-efficacy with regards to behavior change (quitting to smoke) (Warner et al., 2018) and work self-efficacy (though with measurements that were months apart) (Llorens-Gumbau and Salanova-Soria, 2014). Perhaps the process underlying the assumed feedback loop (teacher self-efficacy leading to need-supportive teaching, leading to student intrinsic motivation, leading to teacher self-efficacy) is relatively complex and therefore unfolds on a different (more long term) timescale then the fine-grained lesson-to-lesson timescale which we chose.

In two cases, we did find a relationship over time between student intrinsic motivation and self-efficacy. In short, student intrinsic motivation today can impact on teacher self-efficacy tomorrow, but teacher self-efficacy today does not necessarily translate into need-supportive teaching nor student intrinsic motivation tomorrow. This finding provides some evidence for the hypothesis that student and teacher experiences are indeed intertwined, but the influence might go more from student to teacher than the other way around. The teacher’s experience of mastery (as evidenced by positive student outcomes) is thus an important mechanism for building self-efficacy (and the reverse: unmotivated students have the potential to undermine teachers’ self-efficacy). This is a relevant insight, as the vast majority of the literature on student motivation is about how teachers’ actions impact on the motivation of students. The contrary has been largely uninvestigated.

### 4.1. Limitations and recommendations

One limitation of the current study is that we relied on teachers’ self-report of need-supportive teaching rather than actual observed behavior, which would have provided a more valid picture of need-supportive teaching. The actual need-supportive teaching behaviors are important predictors of student engagement in class (Zeinstra et al., 2023) and in turn, their own perceived motivation (Stroet et al., 2013). We recommend future studies to combine intensive longitudinal self-report data with detailed classroom observations. Furthermore, an interesting addition would be to also include student assessments of need-support, in order to better understand how classroom behavior feeds into student and teacher experiences. Previous research suggests that teacher self-report, student report and observational measures of the quality of teaching and teacher-student interactions provide complementary yet partially unique perspectives on classroom dynamics (Donker et al., 2021).

A second limitation is that we aggregated the students’ motivation scores into one mean motivation score for the whole class. In the future, a multilevel VAR model (Schuurman and Hamaker, 2019; Lafit et al., 2022) where each individual students’ motivation per lesson is taken into account, can provide a more detailed image of how motivation relates to teacher self-efficacy and need-supportive teaching. In addition, an interesting question for future research is whether all students equally impact on teacher’s self-efficacy. Studies on student-specific self-efficacy (Zee et al., 2017) suggest that this might not be the case.

Using students’ self-reported motivation is on the one hand a strength of the study, as this was the first study to systematically link time series of teacher and student self-reports over time. On the other hand, the teacher-perceived student engagement might be a relevant factor that we currently overlooked. If the teacher has the impression that the students are motivated for her classes, that might impact on self-efficacy in the next moment more directly than the actual motivation of the students.

The students and teachers received a small token of appreciation for their participation in the study, which could be seen as a possible limitation because this might have been perceived as an external motivation to keep them engaged in the study. However, this way of keeping participants engaged in an intensive longitudinal study is not uncommon (REFS) and is in our eyes a fair compensation for their time investment. Because the blank submitted responses were left out (see Method section), the results were not biased by students or teachers responding solely for the reward.

A final limitation of the study is the limited number of teachers included. On the one hand this multiple case study design allowed us to collect unique fine-grained data which allowed a more in-depth picture of how teacher self-efficacy develops as a socially nested construct. On the other hand, many more cases like the four presented in the article are necessary to draw conclusions on how common patterns in teacher self-efficacy are amongst teachers, how they relate to other characteristics of teachers (such as years of experience, the school context) and how teacher and student experiences relate to each other.

### 4.2. Implications for educational practice

Low self-efficacy is an important predictor of burn-out (Zhu et al., 2018). Moreover, beginning teachers’ attrition rates are staggering and worrying in relation to school staff shortages around the world. Therefore, schools should be focused on increasing (beginning) teachers’ self-efficacy. There is ample literature on how schools may try to foster self-efficacy with a focus on improving teachers’ skills. Our study shows that the students’ behavior and (intrinsic) motivation may play an important role and is therefore a possibly overlooked area of interest. Two findings are of importance for the support of beginning teachers: the “overspill effect” of student intrinsic motivation from 1 day to the next (if students are motivated today, they will likely also be motivated tomorrow) and relation between student motivation today with teacher self-efficacy tomorrow. Combined, this leads us to advice school leaders to link beginning teachers, or others with low self-efficacy, to (at least some) classes that are in a positive “motivational flow”, as this will increase the likelihood of mastery experiences for the teacher and thereby, impact positively on teachers’ self-efficacy.

## Data availability statement

The raw data supporting the conclusions of this article will be made available by the authors, without undue reservation.

## Ethics statement

The studies involving human participants were reviewed and approved by Ethical Committee Pedagogy and Educational Sciences, University of Groningen. Written informed consent to participate in this study was provided by the participants’ legal guardian/next of kin.

## Author contributions

EK designed the study supervised data collection together with AM, and analyzed the data and wrote the manuscript. JL and AB designed the instruments, recruited participants and collected the data. CA advised on data analysis and co-analyzed the data. All authors contributed to the article and approved the submitted version.

## Funding

This study was funded by NRO (The Netherlands Initiative for Educational Research), file no. 405-17-302.

## Conflict of interest

The authors declare that the research was conducted in the absence of any commercial or financial relationships that could be construed as a potential conflict of interest.

## Publisher’s note

All claims expressed in this article are solely those of the authors and do not necessarily represent those of their affiliated organizations, or those of the publisher, the editors and the reviewers. Any product that may be evaluated in this article, or claim that may be made by its manufacturer, is not guaranteed or endorsed by the publisher.

## References

[ref01] Bandura A. (1977). Self-efficacy: toward a unifying theory of behavioral change. Psychol. Rev. 84:191. 847061 10.1037//0033-295x.84.2.191

[ref02] Blaauw F. J. Van der Gaag M. A. Snell N. R. Emerencia A. C. Kunnen E. S. De Jonge P. (2019). The u-can-act platform: a tool to study intra-individual processes of early school leaving and its prevention using multiple informants. Front. Psychol. 10:1808. doi: 10.3389/fpsyg.2019.01808 31616330 PMC6764284

[ref1] Bouchard S. Gauthier J. Nouwen A. Ivers H. Vallières A. Simard S.. (2007). Temporal relationship between dysfunctional beliefs, self-efficacy and panic apprehension in the treatment of panic disorder with agoraphobia. J. Behav. Ther. Exp. Psychiatry 38, 275–292. doi: 10.1016/j.jbtep.2006.08.002 17157264

[ref2] Chen B. Vansteenkiste M. Beyers W. Boone L. Deci E. L. der Kaap-Deeder V.. (2015). Basic psychological need satisfaction, need frustration, and need strength across four cultures. Motiv. Emot. 39, 216–236. doi: 10.1007/s11031-014-9450-1

[ref03] Daumiller M. Janke S. Hein J. Rinas R. Dickhäuser O. Dresel M. (2021). Do teachers’ achievement goals and self-efficacy beliefs matter for students’ learning experiences? Evidence from two studies on perceived teaching quality and emotional experiences. Learn. Instr. 76:101458.

[ref3] Deci E. L. Eghrari H. Patrick B. C. Leone D. R. (1994). Facilitating internalization: the self-determination theory perspective. J. Pers. 62, 119–142. doi: 10.1111/j.1467-6494.1994.tb00797.x, PMID: 8169757

[ref4] Deci E. L. Ryan R. M. (1985). The general causality orientations scale: self-determination in personality. J. Res. Pers. 19, 109–134. doi: 10.1016/0092-6566(85)90023-6

[ref5] Deci E. L. Vallerand R. J. Pelletier L. G. Ryan R. M. (1991). Motivation and education: the self-determination perspective. Educ. Psychol. 26, 325–346. doi: 10.1080/00461520.1991.9653137

[ref6] Delignières D. Fortes M. Ninot G. (2004). The fractal dynamics of self-esteem and physical self. Nonlinear Dynamics Psychol. Life Sci. 8, 479–510. 15473949

[ref7] Donker M. H. van Vemde L. Hessen D. J. van Gog T. Mainhard T. (2021). Observational, student, and teacher perspectives on interpersonal teacher behavior: shared and unique associations with teacher and student emotions. Learn. Instr. 73:101414. doi: 10.1016/j.learninstruc.2020.101414

[ref8] Dursun O. O. (2019). Pre-service information technology teachers’ self-efficacy, self-esteem and attitudes towards teaching: a four-year longitudinal study. Contemp. Educ. Technol. 10, 137–155. doi: 10.30935/cet.554478

[ref04] Evans D. R. Larsen-Freeman D. (2020). Bifurcations and the emergence of L2 syntactic structures in a complex dynamic system. Front. Psychol. 11:574603. doi: 10.3389/fpsyg.2020.574603 33192875 PMC7658482

[ref05] Houben M. Kuppens P. (2020). Emotion dynamics and the association with depressive features and borderline personality disorder traits: Unique, specific, and prospective relationships. Clin. Psychol. Sci. 8, 226–239.

[ref9] Koopmans M. Stamovlasis D. (2016). Complex dynamical systems in education. Switzerland: Springer International Publishing. 10, 978–973.

[ref10] Koval P. Sütterlin S. Kuppens P. (2016). Emotional inertia is associated with lower well-being when controlling for differences in emotional context. Front. Psychol. 6:1997. doi: 10.3389/fpsyg.2015.01997 26779099 PMC4705270

[ref11] Lafit G. Meers K. Ceulemans E. (2022). A systematic study into the factors that affect the predictive accuracy of multilevel VAR (1) models. Psychometrika 87, 432–476. doi: 10.1007/s11336-021-09803-z, PMID: 34724142

[ref12] Larsen-Freeman D. (2016). Classroom-oriented research from a complex systems perspective. Stud. Second Lang. Learn. Teach. 6, 377–393. doi: 10.14746/ssllt.2016.6.3.2

[ref06] Lauermann F. Berger J. L. (2021). Linking teacher self-efficacy and responsibility with teachers’ self-reported and student-reported motivating styles and student engagement. Learn. Instr. 76:101441.

[ref07] Llorens-Gumbau S. Salanova-Soria M. (2014). Loss and gain cycles? A longitudinal study about burnout, engagement and self-efficacy. Burn. Res. 1, 3–11.

[ref08] Mainhard M. T. Pennings H. J. Wubbels T. Brekelmans M. (2012). Mapping control and affiliation in teacher–student interaction with state space grids. Teach. Teach. Educ. 28, 1027–1037.

[ref13] Molenaar P. C. (2004). A manifesto on psychology as idiographic science: bringing the person back into scientific psychology, this time forever. Measurement 2, 201–218.

[ref14] Murayama K. Goetz T. Malmberg L. E. Pekrun R. Tanaka A. Martin A. J. (2017). Within-person analysis in educational psychology: importance and illustrations. Br. J. Educ. Psychol. Monogr. Ser 12, 71–87. doi: 10.53841/bpsmono.2017.cat2023.6

[ref15] Olivier E. Galand B. Morin A. J. Hospel V. (2021). Need-supportive teaching and student engagement in the classroom: comparing the additive, synergistic, and global contributions. Learn. Instr. 71:101389. doi: 10.1016/j.learninstruc.2020.101389

[ref16] Pfaff B. (2008). VAR, SVAR and SVEC models: implementation within R package vars. J. Stat. Softw. 27. doi: 10.18637/jss.v027.i04

[ref17] Schuurman N. K. Hamaker E. L. (2019). Measurement error and person-specific reliability in multilevel autoregressive modeling. Psychol. Methods 24, 70–91. doi: 10.1037/met0000188, PMID: 30188157

[ref18] Sharma U. Loreman T. Forlin C. (2012). Measuring teacher efficacy to implement inclusive practices. J. Res. Spec. Educ. Needs 12, 12–21. doi: 10.1111/j.1471-3802.2011.01200.x

[ref19] Skaalvik E. M. Skaalvik S. (2007). Dimensions of teacher self-efficacy and relations with strain factors, perceived collective teacher efficacy, and teacher burnout. J. Educ. Psychol. 99, 611–625. doi: 10.1037/0022-0663.99.3.611

[ref20] Steenbeek H. van Geert P. (2013). The emergence of learning-teaching trajectories in education: a complex dynamic systems approach. Nonlinear Dynamics Psychol. Life Sci. 17, 233–267. PMID: 23517608

[ref21] Stroet K. Opdenakker M. C. Minnaert A. (2013). Effects of need supportive teaching on early adolescents’ motivation and engagement: a review of the literature. Educ. Res. Rev. 9, 65–87. doi: 10.1016/j.edurev.2012.11.003

[ref22] Tschannen-Moran M. Woolfolk Hoy A. Hoy W. K. (1998). Teacher efficacy: its meaning and measure. Rev. Educ. Res. 68, 202–248. doi: 10.3102/00346543068002202

[ref23] Van Buuren S. Groothuis-Oudshoorn C. (2011). MICE: multivariate imputation by chained equations in R. J. Stat. Softw. 45. doi: 10.18637/jss.v045.i03

[ref24] von Suchodoletz A. Jamil F. M. Larsen R. A. Hamre B. K. (2018). Personal and contextual factors associated with growth in preschool teachers' self-efficacy beliefs during a longitudinal professional development study. Teach. Teach. Educ. 75, 278–289. doi: 10.1016/j.tate.2018.07.009

[ref25] Wang L. (2021). Exploring the relationship among teacher emotional intelligence, work engagement, teacher self-efficacy, and student academic achievement: a moderated mediation model. Front. Psychol. 12:810559. doi: 10.3389/fpsyg.2021.810559 35046879 PMC8761667

[ref09] Warner L. M. Stadler G. Lüscher J. Knoll N. Ochsner S. Hornung R.. (2018). Day‐to‐day mastery and self‐efficacy changes during a smoking quit attempt: Two studies. Br. J. Health Psychol. 23, 371–386. 29333730 10.1111/bjhp.12293

[ref26] Weikel K. Tomer A. Davis L. Sieke R. (2017). Recovery and self-efficacy of a newly trained certified peer specialist following supplemental weekly group supervision: a case-based time-series analysis. Am. J. Psychiatr. Rehabil. 20, 1–15. doi: 10.1080/15487768.2016.1267051

[ref27] Woolfolk Hoy A. (2021). Teacher motivation, quality instruction, and student outcomes: not a simple path. Learn. Instr. 76:101545. doi: 10.1016/j.learninstruc.2021.101545

[ref28] Zee M. de Jong P. F. Koomen H. M. (2017). From externalizing student behavior to student-specific teacher self-efficacy: the role of teacher-perceived conflict and closeness in the student–teacher relationship. Contemp. Educ. Psychol. 51, 37–50. doi: 10.1016/j.cedpsych.2017.06.009

[ref29] Zee M. Koomen H. M. (2016). Teacher self-efficacy and its effects on classroom processes, student academic adjustment, and teacher well-being: a synthesis of 40 years of research. Rev. Educ. Res. 86, 981–1015. doi: 10.3102/0034654315626801

[ref30] Zee M. Koomen H. M. Jellesma F. C. Geerlings J. de Jong P. F. (2016). Inter-and intra-individual differences in teachers’ self-efficacy: a multilevel factor exploration. J. Sch. Psychol. 55, 39–56. doi: 10.1016/j.jsp.2015.12.003, PMID: 26931066

[ref31] Zeinstra L. Kupers E. Loopers J. de Boer A. (2023). Real-time teacher-student interactions: the dynamic interplay between need supportive teaching and student engagement over the course of one school year. Teach. Teach. Educ. 121:103906. doi: 10.1016/j.tate.2022.103906

[ref32] Zhu M. Liu Q. Fu Y. Yang T. Zhang X. Shi J. (2018). The relationship between teacher self-concept, teacher efficacy and burnout. Teach. Teach. 24, 788–801. doi: 10.1080/13540602.2018.1483913

[ref33] Zivot E. Wang J. (2006). “Vector autoregressive models for multivariate time series” in Modeling financial time series with S-PLUS®. Springerlink, 385–429.

